# Prevalence of smoking in adults with chronic pain

**DOI:** 10.1186/s12971-015-0042-y

**Published:** 2015-07-17

**Authors:** Vwaire J. Orhurhu, Thomas P. Pittelkow, W. Michael Hooten

**Affiliations:** Mayo Clinic College of Medicine, Rochester, MN 55905 USA; Department of Anesthesiology, Division of Pain Medicine, Mayo Graduate School of Medicine, Rochester, MN 55902 USA; Department of Anesthesiology, Division of Pain Medicine, Mayo Clinic College of Medicine, 200 First Street SW, Rochester, MN 55905 USA

**Keywords:** Smoking, Chronic pain, Prevalence, Low back pain, Fibromyalgia, Chronic headache

## Abstract

**Introduction:**

Cigarette smoking is common among adults with chronic pain. The primary objective of this study was to determine the period prevalence of smoking in patients with chronic pain. A secondary objective was to determine the prevalence of smoking among patients with commonly occurring pain diagnoses including fibromyalgia, low back pain, and headache.

**Methods:**

This population study included 5350 patients (1256 smokers, 4094 nonsmokers) admitted to the Mayo Comprehensive Pain Rehabilitation Center from January 1998 through December 2012. Smoking status was determined using a self-report questionnaire.

**Results:**

During the 15 year study period, the overall prevalence of smoking was 23.5 % (95 % CI 22.4 – 24.6). The prevalence of smoking in 2000, 2005, and 2010 was 24.2, 25.7, and 28.3 % respectively. The overall prevalence of smoking in patients with fibromyalgia, low back pain, and headache was 25.2 % (95 % CI 22.8 – 28.3), 22.8 % (95 % CI 21.3 – 25.9), and 21.2 % (95 % CI 17.9 – 24.7), respectively. In a multiple variable logistic model adjusted for age and sex, opioid use was significantly associated with status as a current smoker.

**Conclusion:**

The prevalence of smoking in patients with chronic pain has not declined when compared to the general population. The higher prevalence of smoking was consistently observed in commonly occurring pain diagnoses including fibromyalgia, back pain, and headache. Further research is needed to identify the potential factors that contribute to the high prevalence of smoking in this patient population.

## Introduction

The single greatest preventable cause of death in the United States is cigarette smoking [7]. The health implications of smoking in chronic pain conditions remains complex, but several studies have shown positive associations [4, 11, 20, 26, 28, 31, 41]. For example, smokers report greater pain intensity and a greater number of painful sites compared to nonsmokers [19, 36]. In addition, smokers with chronic pain admitted to specialty pain treatment centers report greater levels of functional impairment compared to nonsmokers [10, 14, 38, 39].

The prevalence of smoking in the general population has declined over the past decade, but is still estimated at 19.3 % [2]. However, the rate of smoking in individuals with chronic pain appears to remain higher compared to the general population [3, 9, 26, 31]. Therefore, the primary aim of this study was to determine the period prevalence of smoking in patients with chronic pain admitted to an interdisciplinary pain treatment center from January, 1998 through December, 2012. A secondary aim was to determine the prevalence of smoking in patients with commonly occurring pain diagnoses including fibromyalgia, chronic low back pain, and headache.

## Methods

### Study participants

This study was approved by the Mayo Foundation Institutional Review Board, and all patients provided prior written consent for use of their medical records for research purposes. Inclusion criteria included admission to the Mayo Comprehensive Pain Rehabilitation Center from January, 1998 through December, 2012, age ≥ 18, and pain duration ≥ 6-months. Exclusion criteria included use of other forms of tobacco besides cigarettes including oral tobacco, cigars, or pipe. Patients who did not indicate smoking status were excluded from study participation. During this time period, 5617 patients with chronic pain were admitted to the pain treatment center; 267 patients (4.9 %) were excluded from study participation due to use of other forms of tobacco and missing data. The population study was comprised of 5350 individuals (1256 smokers, 4094 nonsmokers).

### Study setting

The study setting was a 3-week, outpatient pain rehabilitation program. Descriptions of this treatment setting have been previously reported [14, 17, 18]. In brief, the primary goal of treatment is restoration of physical and emotional functioning. A cognitive-behavioral model serves as the basis for treatment. Patients referred for this type of therapy have generally received medical and surgical care for chronic pain, and experienced incomplete symptomatic relief from conventional treatments including pharmacologic trials, physical therapy, interventional pain procedures, and surgery. Multidisciplinary pain treatment differs from independently prescribed outpatient physical therapy, occupational therapy, and cognitive-behavioral therapy in that patients are involved in all these treatment modalities concurrently on a daily basis throughout the treatment period. Admissions to the rehabilitation program occur on a revolving basis and patients attend 8 h daily for 15 consecutive working days. During the course of treatment, patients are involved in daily physical reconditioning, biofeedback and relaxation training, stress management, chemical health education, activity moderation, and elimination of pain behaviors. Patients are also involved in daily cognitive–behavioral group educational sessions where the aforementioned aspects of pain rehabilitation are addressed.

### Smoking status

Smoking status was assessed upon admission to the pain treatment center using a self-report questionnaire as previously described [14, 17]. Incomplete data were available to categorize never smokers and former smokers into separate groups; therefore, patients identified as either never or former smokers were categorized as nonsmokers. Patients who reported smoking cigarettes upon admission were categorized as smokers.

### Pain diagnosis and pain duration

Upon admission, the primary anatomical site of chronic pain or underlying pain condition (i.e., fibromyalgia) was identified and used as the primary pain diagnosis. This approach for identifying the primary pain diagnosis has been extensively used in our pain treatment program [14, 17, 18]. In addition, the duration of chronic pain was assessed upon admission by self-report and review of the medical record. This method of assessing pain duration has been used extensively in our pain treatment program [14, 17, 18].

### Statistical analyses

The summary statistics for continuous data were reported as an average (± standard deviation; SD), and categorical data were reported as a number (percent). The demographics and clinical characteristics (age, sex, ethnicity, place of residence, pain diagnosis, pain duration) were summarized for smokers and nonsmokers. The comparison between smokers and nonsmokers was made using chi-squared test for categorical variables and univariate analysis of variance for continuous variables. The proportion of smokers and nonsmokers in the year 2000 was compared to the proportion of smokers and nonsmokers in 2005 and 2010 using chi-squared tests. Multiple variable logistic regression analysis was used to assess the potential associations between smoking status (dependent variable) and opioid use (independent variable) in a model adjusted for age and sex. Similarly, multiple variable logistic regression analysis was used to assess the potential associations between smoking status and ethnicity in a model adjusted for age and sex. For purposes of this particular analysis, the Hispanic and African American groups were combined and the “undisclosed” ethnic group was omitted. Two-sided tests were used in all analyses, and the level of significance for all statistical tests was set at *P* < 0.05. All analyses were completed using JMP version 9.0.1 (SAS Institute, Cary, NC).

## Results

### Demographic and clinical characteristics

The population study (*n* = 5350) was comprised of 1256 smokers and 4094 nonsmokers. The total number of women was 3812 and the total number of men was 1538 (Table 1). The majority of patients were married Caucasian women living outside the state of Minnesota. A greater proportion of nonsmokers were currently employed; otherwise, no significant differences in demographic or clinical characteristics based on smoking status were identified.

**Table 1:** Baseline demographic and clinical characteristics of smokers and nonsmokers

Characteristics	Smokers	Nonsmokers	*P* value*
	(*n* = 1256)	(*n* = 4094)	
Age (mean ± SD)	46.3 ± 13.9	45.9 ± 13.5	0.315
Sex (n, %)			0.256
Male	377 (30)	1161 (28)	
Female	879 (70)	2933 (72)	
Ethnicity			0.625
Caucasian	1072 (85.4)	3517 (86.0)	
Hispanic	15 (1.2)	41 (1.0)	
African American	19 (1.5)	78 (1.9)	
Undisclosed	150 (11.9)	458 (11.1)	
Marital status			0.743
Married	786 (63.6)	2546 (63)	
Single/Divorce	449 (36.4)	1487 (39)	
Residence			0.705
Minnesota	503 (40.1)	1663 (40.7)	
Non Minnesota	752 (59.9)	2425 (59.3)	
Years of education	14.9 ± 8.4	14.5 ± 3.0	0.918
Currently employed	196 (15.6)	743 (18.1)	0.042
Pain duration, years	9.7 ± 10.2	9.8 ± 10.6	0.763
Primary pain site			0.738
Fibromyalgia	239 (19.0)	700 (17.1)	
Low back	317 (25.2)	1030 (25.2)	
Headache	117 (9.3)	438 (10.7)	
Generalized	132 (10.5)	439 (10.7)	
Abdominal	97 (7.7)	315 (7.7)	
Neck	92 (7.3)	269 (6.6)	
Lower extremity	92 (7.3)	326 (8.0)	
Face	30 (2.4)	122 (3.0)	
Upper extremity	63 (5.0)	213 (5.2)	
Other	77 (6.2)	242 (5.9)	

*Chi-square for categorical variable, univariate analysis of variance for continuous variable

### Prevalence of smoking in adults with chronic pain

**Fig. 1:**
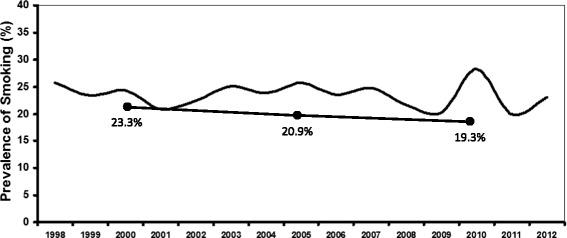
The curved line represents the prevalence of smoking in patients with chronic pain. The straight line indicates the prevalence of smoking in the general population for the years 2000, 2005, and 2010

During the 15 year study period, the overall prevalence of smoking was 23.5 % (95 % CI 22.4 – 24.6). The yearly trend for smoking prevalence ranged from 19.9 to 28.3 % (95 % CI 24.2 – 32.7) (Fig. 1). During the 15 year study period, the prevalence of smoking among Hispanics and African Americans was 26.8 % (95 % CI 15.2 – 38.4) and 19.6 (95 % CI 11.7 – 27.5), respectively. No significant differences in smoking prevalence were observed when smoking status was based on sex and stratified by age (Table 2). Within 5-year intervals starting in 2000, the prevalence of smoking fluctuated but a trend towards increased rates was observed starting in the year 2000 (24.2 %) and extending through 2005 (25.7 %) and 2010 (28.3 %) (Fig. 1). However, no significant difference was observed in the proportion of patients who smoked in the year 2000 compared to 2005 (*P* > .1). Similarly, no significant difference was observed in the proportion of patients who smoked in the year 2000 compared to 2010 (*P* > .1).

**Table 2:** Prevalence of smoking based on sex and stratified by age

Characteristics	Prevalence (95 % CI*)	Prevalence (95 % CI)	Prevalence (95 % CI)
	Men (*n* = 1538)	Women (*n* = 3812)	Total (*n* = 5350)
Overall	24.5 (22.4–26.7)	23.1 (21.7–24.4)	23.5 (22.4–24.6)
Age group (yrs)			
18 – 24	22.2 (14.0–33.1)	26.2 (20.9–32.3)	25.2 (20.8–30.2)
25 – 44	25.5 (22.1–29.3)	22.2 (20.2–24.4)	23.1 (21.4–24.9)
45 – 64	23.2 (20.2–26.5)	22.8 (20.9–25.0)	23.0 (21.3–24.7)
≥ 65	28.1 (21.3–36.0)	25.3 (21.1–29.9)	26.0 (22.5–29.9)

*CI = confidence interval

### Prevalence of smoking in diagnostic subgroups

During the 15 year study period, the overall prevalence of smoking in patients with fibromyalgia, low back pain, and headache was 25.2 % (95 % CI 22.8 – 28.3), 22.8 % (95 % CI 21.3 – 25.9), and 21.2 % (95 % CI 17.9 – 24.7), respectively.

### Associations between smoking status, opioid use, and ethnicity

In a logistic regression model adjusted for age and sex, opioid use was significantly associated with status as a current smoker (odds ratio = 1.4, 95 % CI 1.2 to 1.6, *P* = < .001 ). In a logistic regression model adjusted for age and sex, no significant association was found between ethnicity and smoking status (odds ratio = 1.0, 95 % CI .99 – 1.1, *P* = .704).

## Discussion

The period prevalence of smoking in this group of patients with chronic pain admitted to an outpatient pain rehabilitation program over a 15-year period was 23.5 %. No significant differences in the prevalence of smoking was observed among subgroups of patients diagnosed with fibromyalgia, low back pain, or headache. However, a significant association was observed between opioid use and status as a current smoker in a multiple variable regression model adjusted for age and sex.

The prevalence of smoking in the general population in 2000, 2005, and 2010 was 23.3, 20.9, and 19.3 %, respectively [2, 6]. For the same time periods, the prevalence of smoking in our sample of adults with chronic pain was 24.2, 25.7, and 28.3 %, respectively. Although fluctuations in the prevalence of smoking occurred during the time period of the study, the increasing trend observed in our study compared to the decreasing trend in the general population could be due, in part, to clinical characteristics unique to patients with chronic pain including concomitant use of opioids. In the current study, opioid use was significantly associated with status as a current smoker. This is an important because we have previously reported that smokers were more likely to use opioids compared to former and never smokers [15, 16]. In addition, smokers were more likely to consume greater quantities of opioids due, in part, to use of greater dosages by men [15]. The association between smoking and opioid dose occurred independent of key demographic and clinical characteristics including age, marital status, years of education, employment status, pain duration, and pain severity [13, 16]. Previous studies also suggest that it may be more difficult for smokers receiving long-term opioid therapy to quit smoking [14, 17, 18]. These clinical observations are supported by preclinical studies that suggest the antinociceptive effects of nicotine and morphine are linked, and that morphine-related antinociception is influenced by activation of supraspinal nicotinic acetylcholine receptors [30, 33, 34].

Although no significant association was found between ethnicity and smoking status in our study, the prevalence of smoking among Hispanic and African Americans was mixed when compared to the general population. For example, in the general population the prevalence of smoking among Hispanics in 2000, 2005 and 2010 was 18.6, 16.2, and 12.5 %, respectively [6, 7]. During the 15 year time period of our study, the prevalence of smoking among Hispanic adults with chronic pain was 26.8 % which was higher compared to the general population. Conversely, in the general population the prevalence of smoking among African Americans in 2000, 2005, and 2010 was 23.2, 21.5, 18.1 %, respectively [6, 7]. During the 15 year time period of our study, the prevalence of smoking among African Americans was 19.6 %. In previous studies from our pain treatment center, the prevalence of smoking among a small group of Hispanic (*n* = 26) and African American (*n* = 22) adults with chronic pain was 11.5 and 31.8 %, respectively [13, 15, 16]. These mixed data derived from small samples suggest that larger ongoing studies are needed to further investigate the prevalence of smoking among Hispanic and African Americans with chronic pain.

The three most commonly occurring pain diagnoses in our study sample were fibromyalgia, low back pain, and chronic headache, and the observed prevalence of smoking among patients in these diagnostic groups extends and confirms the observations of previous studies. The prevalence of smoking among patients with fibromyalgia during the 15 year study period was 25.2 %. In previous studies, the prevalence of smoking among patients with fibromyalgia ranged from 9.8 to 25.5 % [23, 25, 27, 39, 40]. Greater impairments in pain-related functioning were observed among patients with fibromyalgia who smoke [39]. Patients in our study were admitted to a 3-week outpatient pain rehabilitation program that specifically treated individuals with pain-related impairments in functioning which could partly explain the high prevalence of smoking in our patients with fibromyalgia. Smoking is widely recognized to be a risk factor for chronic low back pain [21, 32]. In previous studies, the prevalence of smoking in this patient population ranged from 16 % to as high as 40 % [5, 22, 24, 36]. The prevalence of smoking during the 15 year study period in our group of patients with low back pain (22.8 %) was consistent with these previously reported observations. Finally, the prevalence of smoking among patients with chronic headache, including migraine, ranged from 3 % to as high as 25 to 28 % in previous studies [1, 8, 29, 35]. During the 15 year study period, our observed prevalence of 21.2 % was consistent with these previous reports, but direct comparisons are limited by the diagnostic heterogeneity that exists among our headache patients.

This study has limitations. First, the majority of study participants were Caucasian women residing in the United States, and all study participants were specifically referred for pain rehabilitation at a tertiary medical center. This is consistent with the referral pattern of our pain treatment program [12, 15], but it could limit the generalization of the study findings to other populations of adults with chronic pain. However, the clinical characteristics of patients admitted to the pain program were comparable to those of a random sample of community adults with chronic pain derived from the catchment area of our medical center [37]. Second, patients were assigned to 1 of 10 broadly defined diagnostic groups upon admission to the pain rehabilitation program, but the specific subtype of pain (e.g., neuropathic, nociceptive) was not assessed. This could have influenced the prevalence of smoking in the defined diagnostic subgroups. Third, never smokers and former smokers were categorized as nonsmokers. We have previously reported that significant differences exist between never and former smokers with chronic pain. More specifically, former smokers were older, reported longer pain duration, and were more likely to use daily prescription opioids compared never smokers [13, 15, 16]. The categorization of never and former smokers as nonsmokers could explain the absence of significant group differences in the baseline demographic and clinical characteristics between smokers and nonsmokers as reported herein. Fourth, the number of cigarettes smoked daily was not assessed. However, we have previously reported that smokers admitted to our pain treatment program consume 14 to 18 cigarettes daily [15–17].

In summary, the prevalence of smoking in patients with chronic pain has not declined when compared to the general population. The high prevalence of smoking was consistently observed in commonly occurring pain diagnoses including fibromyalgia, back pain, and headache. The high prevalence of smoking among patients with chronic pain could be related, in part, to unique factors that exist in this patient population including concurrent opioid use.
